# Supplementary material for: Ethanol Disrupts the Protective Crosstalk Between Macrophages and HBV-Infected Hepatocytes
Source: Biomolecules. 2025 Jan 3;15(1):57. doi: 10.3390/biom15010057 (PMC11761873; doi:10.3390/biom15010057)
Supplement: Supplementary file 1 [file biomolecules-15-00057-s001.zip › biomolecules-3249499-supplementary.pdf]

Supplement (original blots)

A. Fig 5. Original Western Blots

STAT1: 3 experiments

1A- Original blot shown in the paper

2A- Experiments 2 and 3 (on the same blot)

B-actin: 3 experiments

3A- Original blot shown in the paper

4A- Experiment 2 and 3 (on the same blot)

PSTAT1: 3 experiments

5A- Original blot shown in the paper (under two different exposures to make the ladder more visible)

6A- Experiments 2 and 3 (on the same blot)

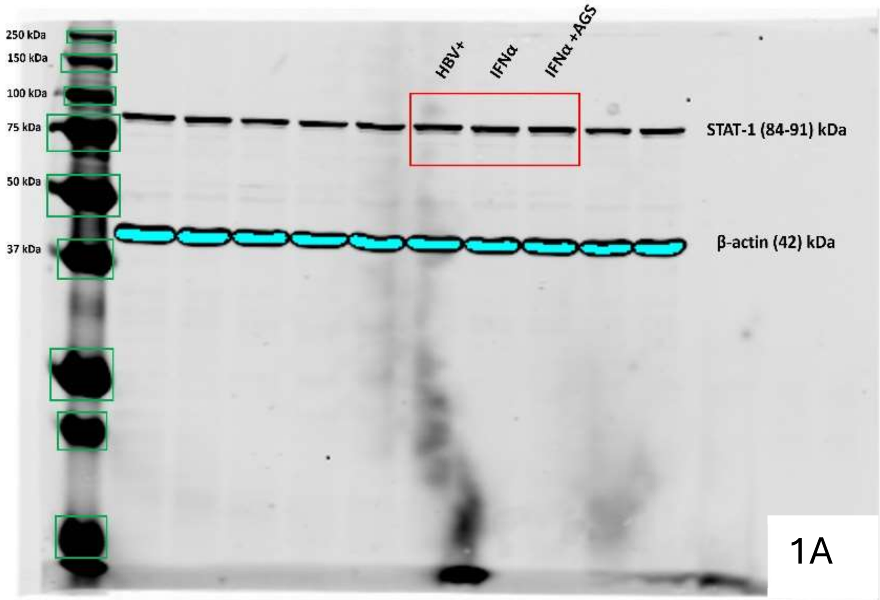

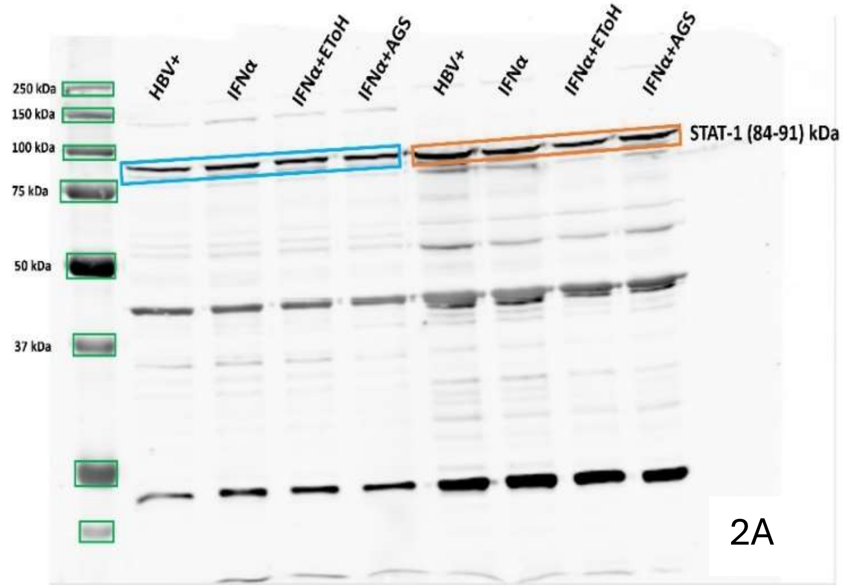
250 kDa
150 kDa
100 kDa
75 kDa
50 kDa
37 kDa
HBV+
IFNα
IFNα+EToH
IFNα+AGS
HBV+
IFNα
IFNα+EToH
IFNα+AGS
STAT-1 (84-91) kDa
2A

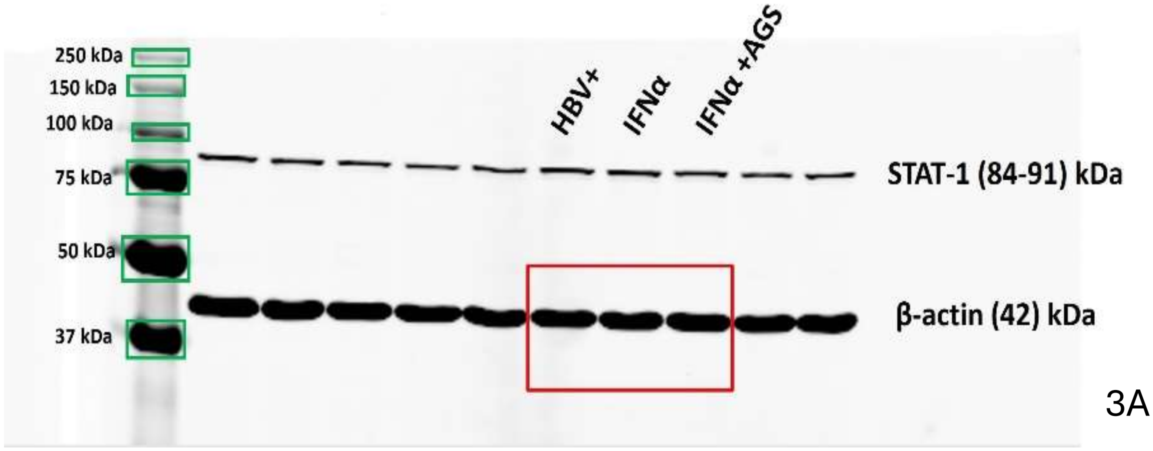
250 kDa
150 kDa
100 kDa
75 kDa
50 kDa
37 kDa
HBV+
IFNα
IFNα +AGS
STAT-1 (84-91) kDa
β-actin (42) kDa
3A

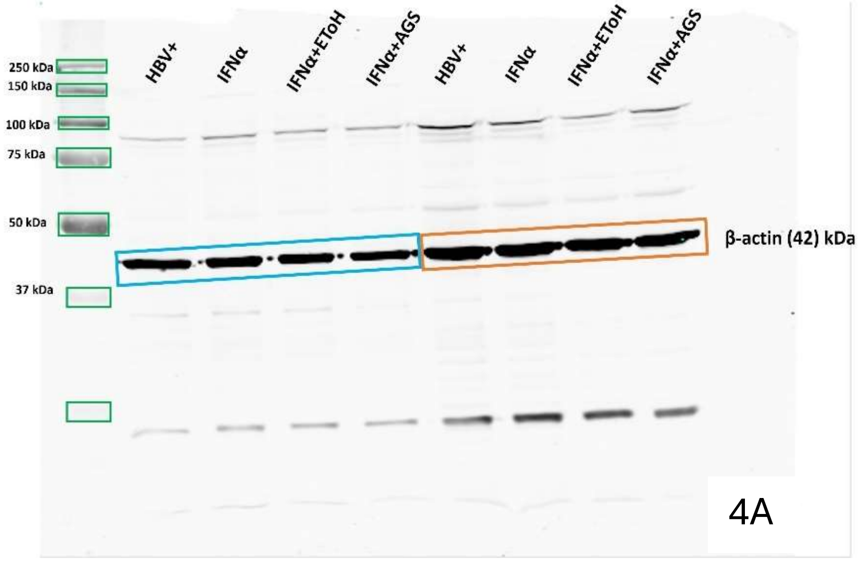
250 kDa
150 kDa
100 kDa
75 kDa
50 kDa
37 kDa
HBV+
IFNα
IFNα+EToH
IFNα+AGS
HBV+
IFNα
IFNα+EToH
IFNα+AGS
β-actin (42) kDa
4A

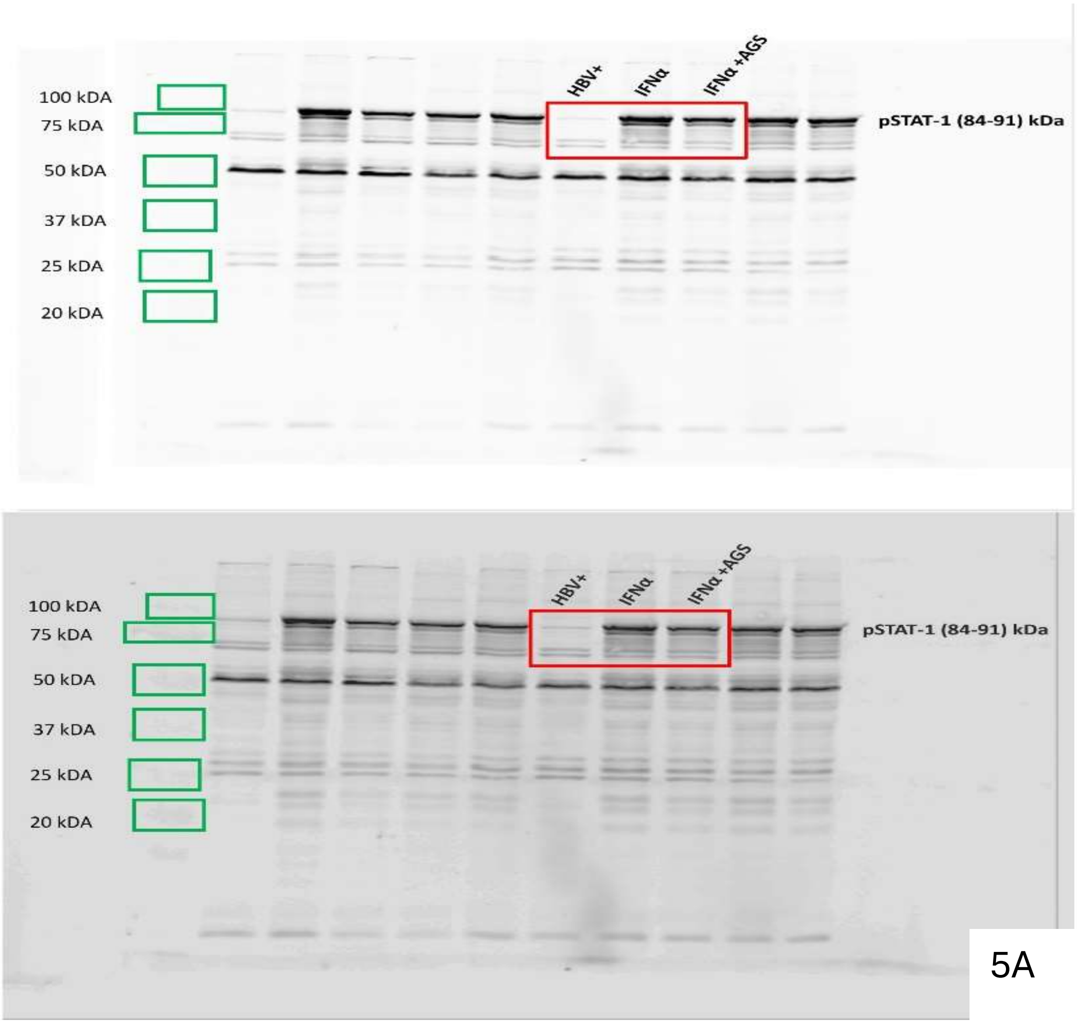
100 kDA
75 kDA
50 kDA
37 kDA
25 kDA
20 kDA
HBV+
IFNα
IFNα +AGS
pSTAT-1 (84-91) kDa
100 kDA
75 kDA
50 kDA
37 kDA
25 kDA
20 kDA
HBV+
IFNα
IFNα +AGS
pSTAT-1 (84-91) kDa

5A

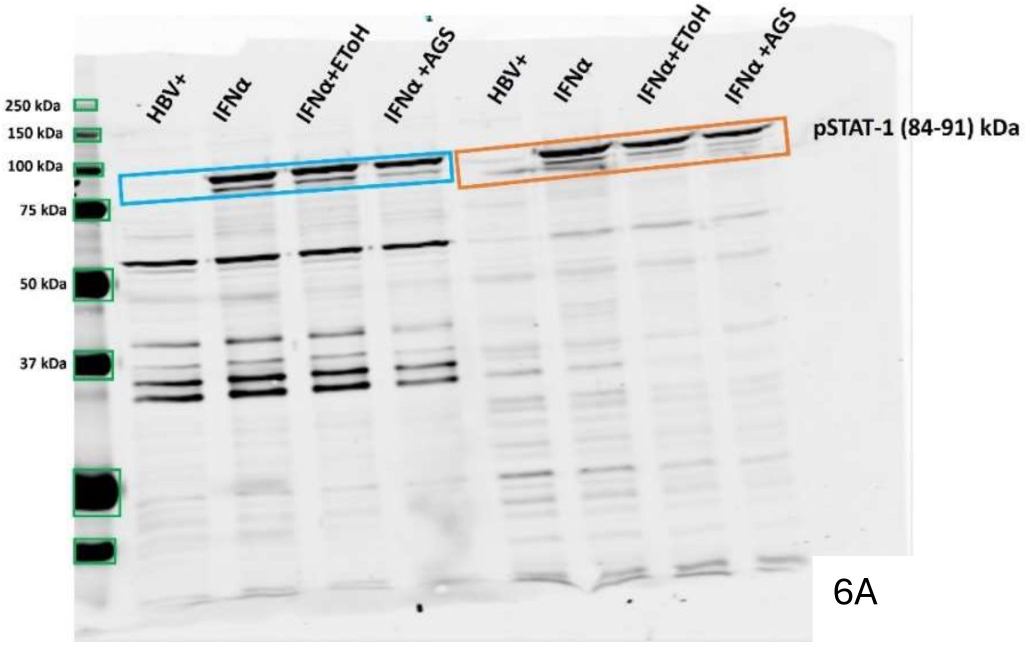
250 kDa
150 kDa
100 kDa
75 kDa
50 kDa
37 kDa
HBV+
IFNα
IFNα+EToH
IFNα +AGS
HBV+
IFNα
IFNα+EToH
IFNα +AGS
pSTAT-1 (84-91) kDa

6A

B. Fig 7. Original Western Blots

IP (Methyl arginine IP-STAT1): 3 experiments
- 1B- Blot shown in the paper
- 2B- Experiments 2 and 3

P-STAT1: 3 experiments
- 3B- Blot shown in the paper
- 4B- Experiments 2 and 3 (on the same blot)

STAT1: 3 experiments
- 5B- Experiment shown in the paper
- 6B- Experiments 2 and 3

STAT1 Input: 3 experiments
- 7B- Blot shown in the paper
- 8B- Experiments 2 and 3

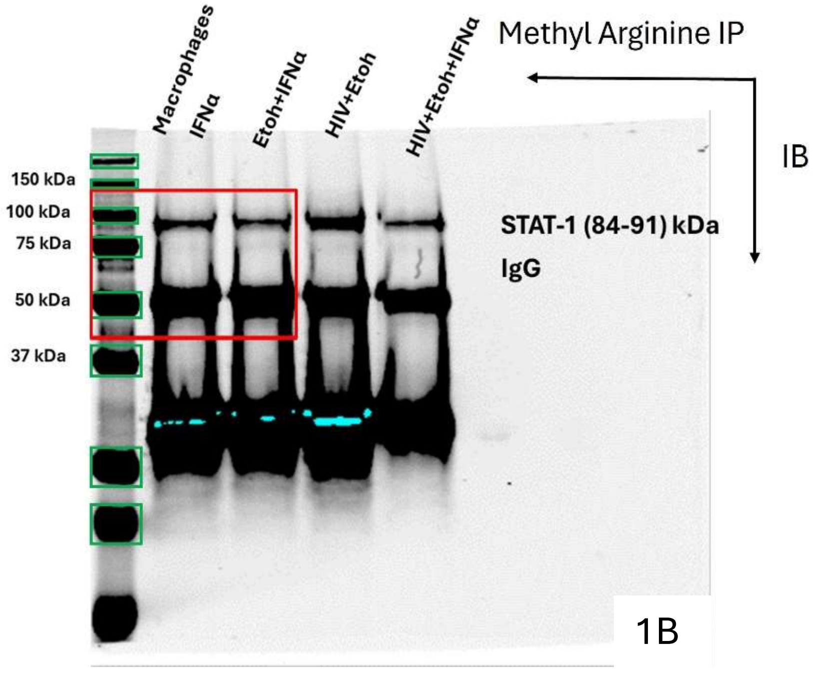

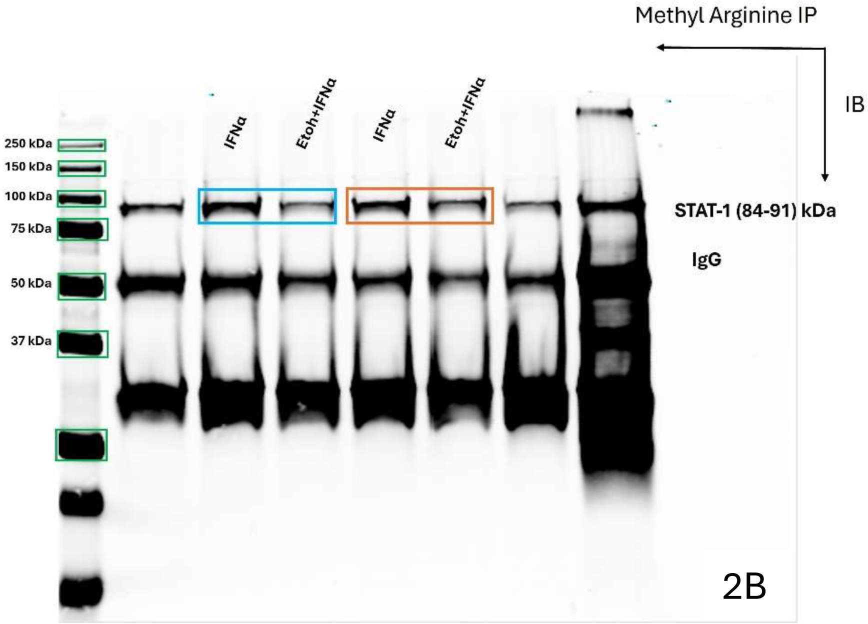
Methyl Arginine IP
IB
IFNα
Etoh+IFNα
IFNα
Etoh+IFNα
250 kDa
150 kDa
100 kDa
75 kDa
50 kDa
37 kDa
STAT-1 (84-91) kDa
IgG

2B

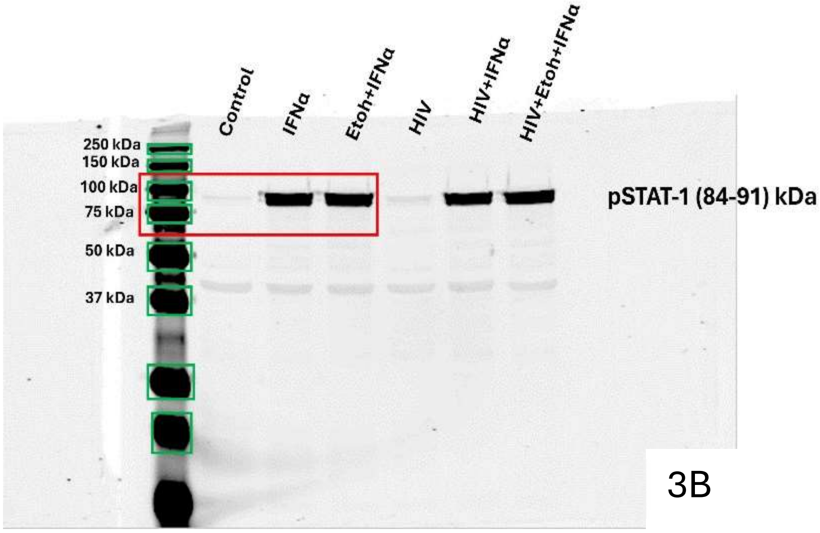
Control
IFNα
Etoh+IFNα
HIV
HIV+IFNα
HIV+Etoh+IFNα
250 kDa
150 kDa
100 kDa
75 kDa
50 kDa
37 kDa
pSTAT-1 (84-91) kDa

3B

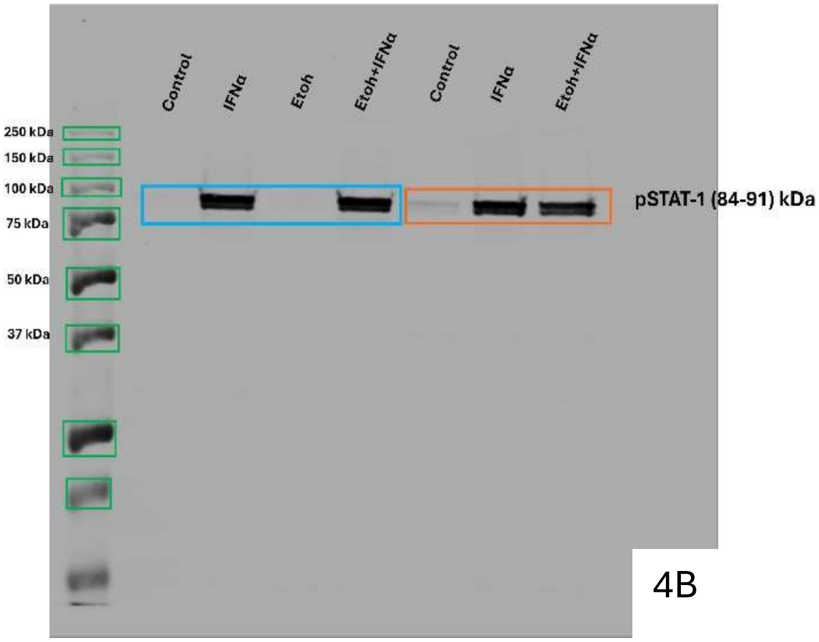
Control
IFNα
Etoh
Etoh+IFNα
Control
IFNα
Etoh+IFNα
250 kDa
150 kDa
100 kDa
75 kDa
50 kDa
37 kDa
pSTAT-1 (84-91) kDa

4B

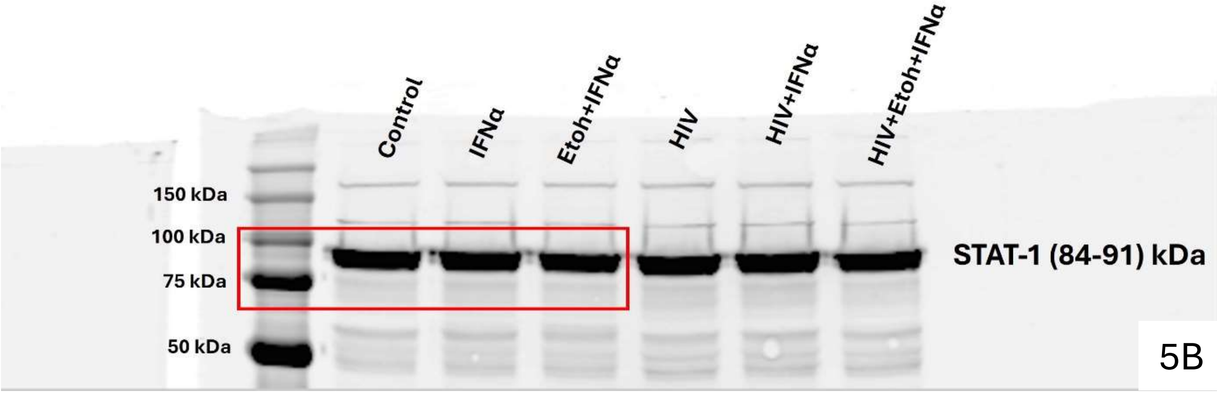
Control
IFNα
Etoh+IFNα
HIV
HIV+IFNα
HIV+Etoh+IFNα
150 kDa
100 kDa
75 kDa
50 kDa
STAT-1 (84-91) kDa

5B

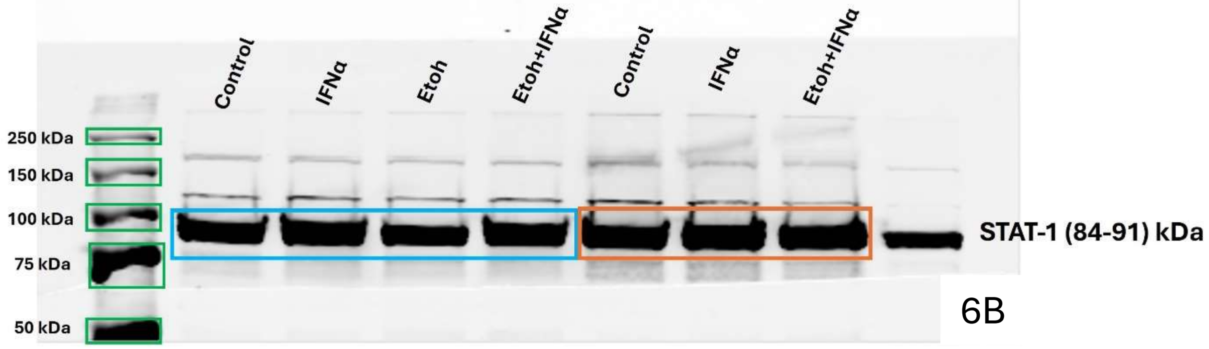
Control
IFNα
Etoh
Etoh+IFNα
Control
IFNα
Etoh+IFNα
250 kDa
150 kDa
100 kDa
75 kDa
50 kDa
STAT-1 (84-91) kDa

6B

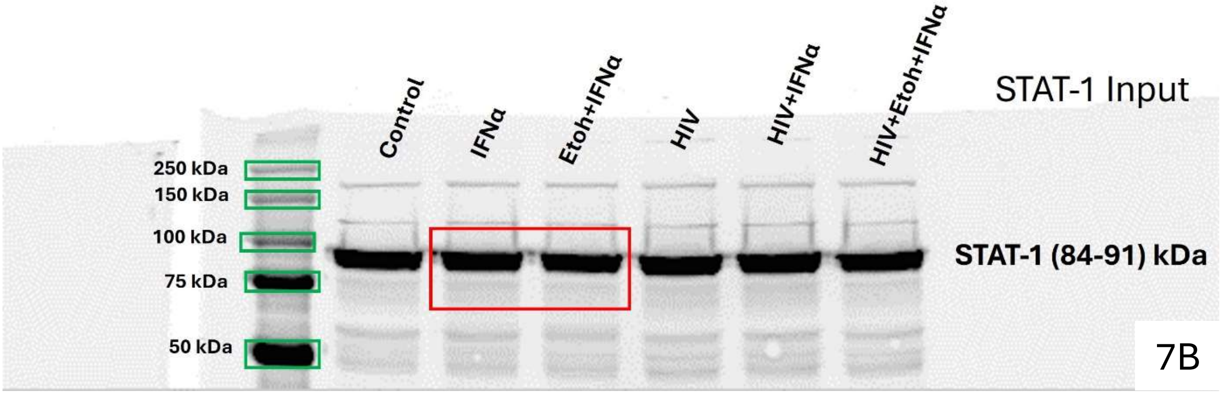
STAT-1 Input
Control
IFNα
Etoh+IFNα
HIV
HIV+IFNα
HIV+Etoh+IFNα
250 kDa
150 kDa
100 kDa
75 kDa
50 kDa
STAT-1 (84-91) kDa

7B

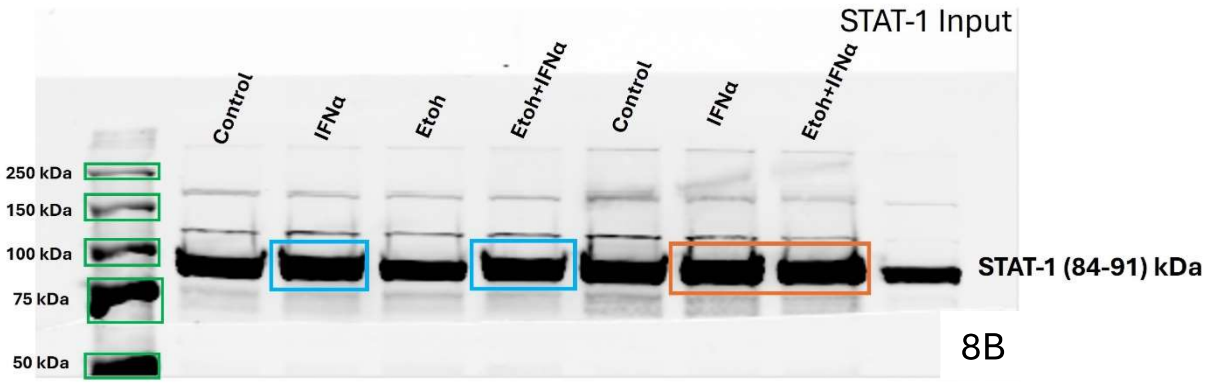
STAT-1 Input
Control
IFNα
Etoh
Etoh+IFNα
Control
IFNα
Etoh+IFNα
250 kDa
150 kDa
100 kDa
75 kDa
50 kDa
STAT-1 (84-91) kDa

8B
